# Impact of structural prior knowledge in SNV prediction: Towards causal variant finding in rare disease

**DOI:** 10.1371/journal.pone.0204101

**Published:** 2018-09-28

**Authors:** Vasundhara Dehiya, Jaya Thomas, Lee Sael

**Affiliations:** 1 Department of Computer Science, State University of New York Korea, Incheon, Republic of Korea; 2 Department of Computer Science, Stony Brook University, Stony Brook, NY, United States of America; 3 Department of Computer Science and Engineering, Seoul National University, Seoul, Republic of Korea; CNR, ITALY

## Abstract

Can structural information of proteins generate essential features for predicting the deleterious effect of a single nucleotide variant (SNV) independent of the known existence of the SNV in diseases? In this work, we answer the question by examining the performance of features generated from prior knowledge with the goal towards determining the pathogenic effect of rare variants in rare disease. We take the approach of prioritizing SNV loci focusing on protein structure-based features. The proposed structure-based features are generated from geometric, physical, chemical, and functional properties of the variant loci and structural neighbors of the loci utilizing multiple homologous structures. The performance of the structure-based features alone, trained on 80% of HumVar-HumDiv combination (HumVD-train) and tested on 20% of HumVar-HumDiv (HumVD-test), ClinVar and ClinVar rare variant rare disease (ClinVarRVRD) datasets, showed high levels of discernibility in determining the SNV’s pathogenic or benign effects on patients. Combined structure- and sequence-based features generated from prior knowledge on a random forest model further improved the F scores to 0.84 (HumVD-test), 0.75 (ClinVar), and 0.75 (ClinVarRVRD). Including features based on the difference between wild-type in addition to the features based on loci information increased the F score slightly more to 0.90 (HumVD-test), 0.78 (ClinVar), and 0.76 (ClinVarRVRD). The empirical examination and high F scores of the results based on loci information alone suggest that location of SNV plays a primary role in determining functional impact of mutation and that structure-based features can help enhance the prediction performance.

## Introduction

Rare diseases have been known to affect over 350 million people worldwide. There are over 7,000 different rare diseases and around 80% are due to genetic factors [1]. Compared to common complex diseases, rare diseases are often heterogeneous, caused by rare variants (minor allele frequency inferior to 1%), and are often hereditary [2, 3]. Many rare diseases can be cared for if diagnosed early and optimally managed. However, fast and accurate diagnosis is difficult in rare disease cases due to the rarity of events. A quick and non-statistical method for identifying a causal variant of rare diseases through variant prioritization forms the basis for fast and accurate diagnosis as well as for providing alternative treatment suggestions [4, 5].

The informatics approach for prioritizing single nucleotide variation (SNV) considers two factors: the importance of the SNV loci (prior) and the degree of difference between the wild-type and the variant. The importance of the SNV loci can be computed as a prior knowledge independent of the difference between wild-type and variant. This means that importance of the loci, i.e., prioritizing SNV loci, can be trained and learned in the preprocessing step independent of disease types, allowing for quick postprocessing when the variant type becomes known. On the other hand, the severity of the SNV in diseases relies on the information of the nucleotide variation compared to wild-type and often requires known cases of the variation in diseased patients.

Features used for pathogenic SNV prediction methods are generally sequence-based only or sequence and structure combined. Although there have been several extensive studies for sequence-based features due to their abundance and ease of usage, there has not been a rigorous and extensive examination of structure-based features in the causal mutation predictions. Many structure focused methods are limited to specific disease type [6] or are more of an annotator [7]. Ones that utilize protein structure in addition to sequence information considers the structure-based features as not essential [8–10]. However, structural features have a high potential for discerning pathogenic effects.

Several works show that mutations on functional and structurally stabilizing sites are strongly related to impaired protein function [11, 12] that results in diseases. Disease-causing P53 structures contain amino acid alterations at key sites that are important for maintaining structural integrity [13, 14]. Also, amino acid alterations that disrupt the thermodynamic and kinetic stability of the TTR proteins, causing them to unfold and hence accumulate to form amyloid fibrils [15], are known to cause many amyloid diseases. Mutations at binding sites in proteins can disrupt the protein’s function as they lead to changes in binding affinity between the protein and other biomolecules. The affected binding targets may be ions, ligands, other proteins, DNAs or RNAs. P53 structures that contain amino acid alterations in the DNA binding region changes the binding affinity of the protein to DNA [16, 17]. A study of somatic mutations in DNA methyltransferase gene DNMT3A in acute monocytic leukemia found mutations in DNA binding, cofactor binding, protein-protein interaction sites and Histone H3 peptide binding sites [18]. These mutations are crucial as they prevent protein complex formation thereby leading to impaired gene function. Also, rare mutations in DNA-binding site in POT1 protein have been identified as causal for cutaneous malignant melanoma as these mutations changed protein folding and binding [19]. Another study identified SNV in the pre-mRNA binding region in splicing factor SF3B1 gene which is known to cause chronic lymphocytic leukemia [20]. These studies highlight the importance of structure-based information in causal mutation analysis in disease which we will focus on through structure-based features extraction.

The goal of this work is to show that well-curated structure-based features provide added value when combined with sequence-based features in the prioritization of SNV loci. We aim to show that loci at which the SNV occurred, even without the difference between wild-type information, is useful in identifying deleterious SNVs in diseases. Also, we aim to provide evidence that structure-based features act as an important determinant for deleteriousness of loci, especially in rare disease. More specifically, the four contributions of the paper are as follows. First, we have developed novel structural features, multi-structural features and structural-neighborhood features. Second, we have extensively tested the performance of structure-based features in determining pathogenic effects of point mutations. Third, we have compared the performance of loci specific features, using various machine learning methods, and state-of-the-art methods. Finally, we have demonstrated the soundness of generated features for rare variant rare disease causal mutation predictions.

## Materials and methods

### Data sets

We generated three data sets, i.e., HumVar-HumDiv (HumVD), ClinVar, ClinVar Rare Variant Rare Disease (ClinVarRVRD) dataset, to compare and test the performance of proposed features. Initially, SNV information, including loci, along with the known functional impact of the SNV was extracted from HumDiv, HumVar [8], and ClinVar databases [21], separately.

We combined the HumVar and HumDiv datasets, as suggested in the documents of PolyPhen2 [8] and available at (http://genetics.bwh.harvard.edu/pph2/dokuwiki/downloads), to generate our HumVD dataset. We used the same SNV classification as PolyPhen2 (Benign = Neutral and Pathogenic = Deleterious). Cases of conflicting labels between HumVar and HumDiv were excluded. Also, we made sure that the protein names and mutation positions of the selected SNVs are unique to avoid bias. After feature generation, 10,140 cases out of 10,583 cases remained, consisting of 3,256 Benign and 6,884 Pathogenic variants. We used 80% of this dataset (2591 Benign and 5521 Pathogenic) for training the model (HumVD-train) and used remaining 20% (665 Benign and 1363 Pathogenic)for testing the model (HumVD-test).

To test whether the model generalizes well, we selected separate set of test cases from ClinVar. The ClinVar database contains a more up-to-date and carefully curated archive of human variations and phenotypes [21]. From the initial database of 93,946 SNV entries retrieved on Jan. 2016, we selected 9,286 SNVs with high confidence review status (2 through 4). The review status 2 is ‘SNV submitted with criteria provided, two or more submitters with no conflicts’; status 3 is ‘reviewed by an expert panel’; status 4 is ‘practice guideline.’ From the 9,286 SNVs, we selected 6,446 loci labeled as either likely benign or benign as Benign and likely pathogenic or pathogenic as Pathogenic. Cases with conflicting labels and overlaps with the HumVD dataset were removed. As the final step, SNVs without protein structure mapping was removed resulting in a small set of 437 SNV loci, with 285 pathogenic and 152 benign labels in 101 genes.

We also constructed rare variant rare disease (ClinVarRVRD) dataset to test how well the model performs on rare disease cases. ClinVarRVRD dataset is a subset of the ClinVar dataset constructed by filtering out non-rare variants identified via Ensembl Variant Effect Predictor tool [22] to exclude common variants with minor allele frequency (MAF) greater than 1% in the 1000 genomes Phase 1 combined population. The ClinVar rare variants were further filtered to make sure they are associated with rare diseases. That is, we considered SNV to be associated with a rare disease if one or more of the diseases label in ClinVar belonged to the Global Genes [1] rare diseases list (6,537 rare diseases as of August 6, 2017). After the two filtering process, 280 rare variants rare disease associated SNVs consisting of 117 benign SNVs and 163 pathogenic SNVs remained.

Table 1 summarizes the dataset. HumVD-train, HumVD-test, ClinVar, and ClinVarRVRD are provided in S1, S2, S3 and S4 Files, respectively.

**Table 1:** Dataset statistics. The number of benign and pathogenic variants for each dataset are listed.

Dataset	Benign	Pathogenic	Total
HumVD (all)	3256	6884	10140
HumVD-train (80%)	2591	5521	8112
HumVD-test (20%)	665	1363	2028
ClinVar	152	285	437
CinVarRVRD	117	163	280

### Features to loci mapping

**Fig 1:**
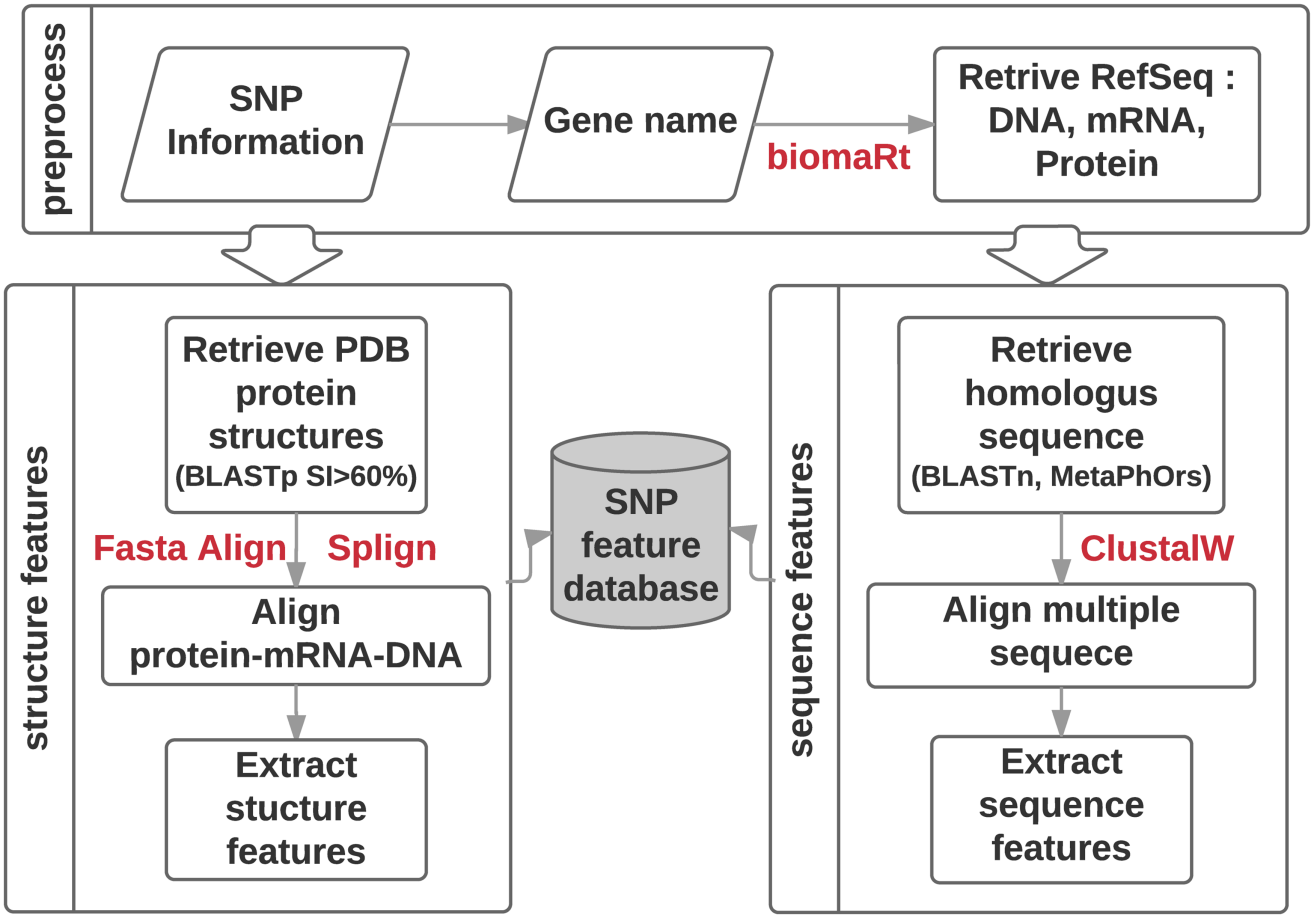
Loci specific feature extraction pipeline.

To extract loci specific features, we retrieved reference DNA, mRNA, and protein sequences of the corresponding loci. To do this, we identified genes associated with the SNV in the DNA loci, represented as a chromosome number and offset value pair, and used biomaRt [23] library to retrieve reference sequences for DNA (DNA-RefSeq), mRNA (NM-RefSeq) and protein amino acid sequence (NP-RefSeq) from Ensembl database [24] corresponding to genome reference HG38 for each dataset. DNA, mRNA, and protein sequences are then aligned to map the features back the SNV loci. Fig 1 shows the steps involved in the feature generation process utilizing the retrieved sequences. Detailed steps are described in detail in the following feature extraction process description.

### Extracting structure-based features

Given an SNV locus, we extracted structure-based feature by first retrieving the protein structures for the locus. To do this, we retrieved a list of protein structures (PDBIDs) of corresponding NP-RefSeq using protein BLAST [25] with homology threshold of 60% sequence identity. If structures were found, the corresponding Protein Data Base (PDB) files were downloaded from RSCB [26] database. From the PDB structures, PDB sequences were directly extracted and pair-wise global aligned using dynamic programming based aligner, i.e., FASTA align [27], to the protein reference sequence (NP-RefSeq). Corresponding reference mRNA sequence (NM-RefSeq) of the NP-RefSeq were then aligned to reference DNA (NC-RefSeq) using Splign [28], which allows alignment even in the presence of splice sites. Finally, the structure-based features were generated and mapped to the SNV locus.

#### Structural neighborhood

**Fig 2:**
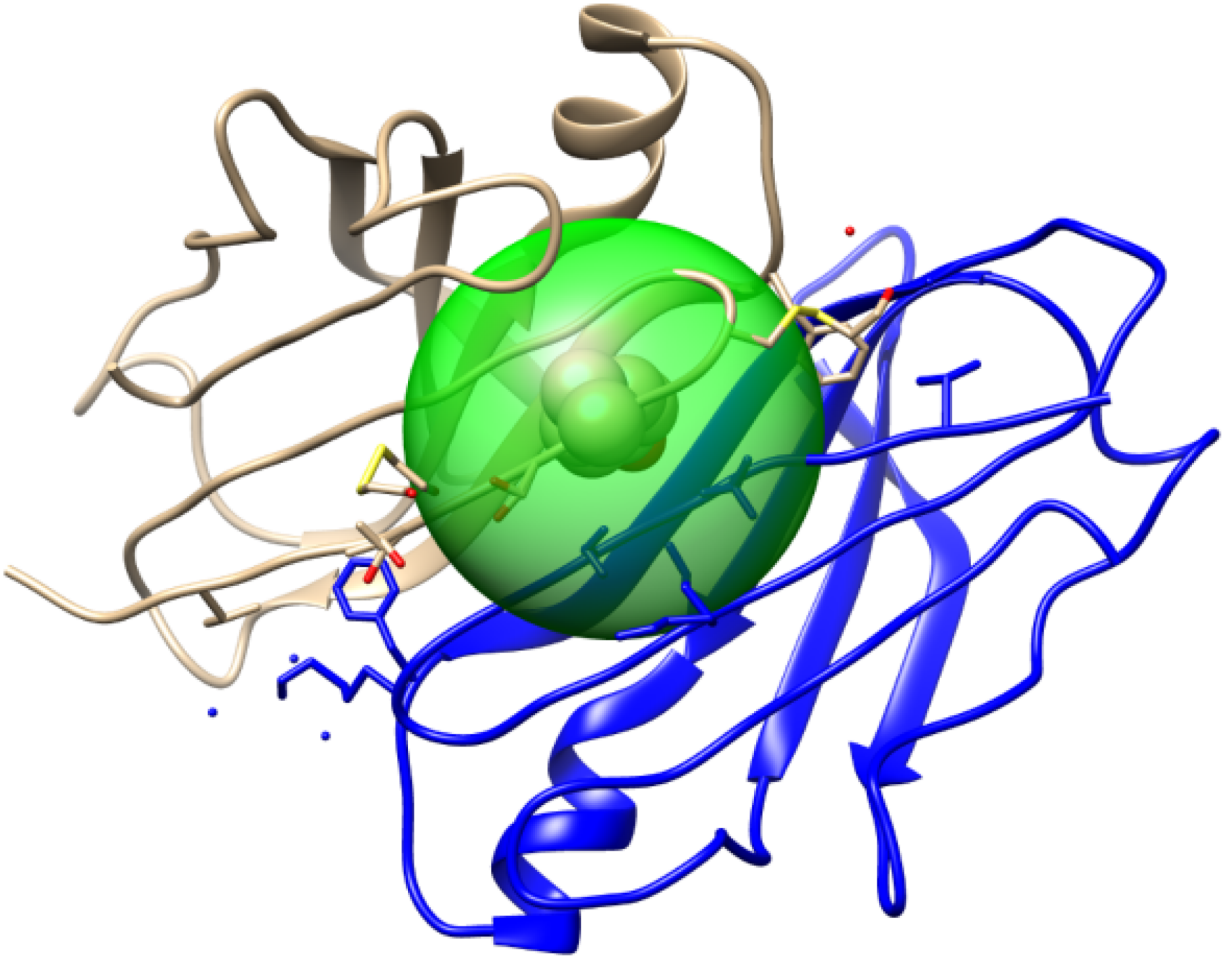
Structural neighborhood. Green sphere depicts a radius of 9Å centered at 116.A of PDBID:1IJN structure. All amino acids residues found within this region are considered as neighbors of the query SNV loci, 116.A.

Introduction of features generated from structural neighbors is one of the novel contributions of our proposed method. We defined the structural neighborhood as the set of amino acid residues that lie within a specified radius of the physical structure of the protein centered around the query SNV loci. Based on the relative Cartesian coordinates of the amino acids in the PDB files, we considered the neighborhood as all the residues located within the Euclidean distance of 9 Angstroms (Å), as shown in Fig 2. We have tested distance between 5 to 15Å and 9Å provided adequated information without involving too many amino-acids. We took summaries of the feature values calculated for neighboring amino acids as the neighborhood features. Also, the number of residues in the neighborhood (*nNum*) was also included as one of the neighborhood features. Since neighborhood information was extracted from a structure, all the neighborhood features were considered as structure-based features.

#### Structure-based features

Another novelty of our structure-based feature is the extraction of information from multiple homologous structures. The reason why we have decided to take information from multiple structures is that a PDB structure is only a static snapshot of the actual protein and the functional information such as binding event may exist in one structure while not on another depending on the experimental settings, thus examining all homologous structures is important. Again, the eleven structure-based features depend on the location of the SNV and not on the actual difference between wild-type and are considered prior knowledge. The list of structural features are shown in Table 2 and the details of the structure-based feature extraction process are provided in the S1 Appendix.

**Table 2:** List of structure- and sequence-based features. The rank represents the importance of individual features obtained by 5-fold cross validation on the HumVar-HumDiv training data (Size = 8,112) using Random Forest for attribute evaluation using weighted F score as evaluation metric.

Name	Description	Rank
**Structure-based features**		
KDmean	mean KD hydrophobicity value	2
RSAmax	maximum residual solvent accessibility value	4
nRSA	mean residual solvent accessibility of structural neighbors	5
nNum	number of amino acids whin structural neighborhood	6
Bstddev	standard deviation of B factors	7
nB	mean B factor of structural neighbors	8
nKD	mean KD hydrophobicity of structural neighbors	10
nSC	mean sequence conservation of structural neighbors	13
nBinding	number of binding site types in neighborhoods	14
Binding	number of binding site types	15
Mapreg	whether SNV locus is within core region of phi-psi Ramachandran map (PolyPhen2)	16
**Sequence-based features**		
PSIC	PSIC score of wild type amino acid (PolyPhen2)	1
Nobs	number of amino acid observed at the substitution position in the multiple alignment (PolyPhen2)	3
MinDJxn	distance of SNV locus from closest exon/ intron junction (PolyPhen2)	9
CodPos	position of SNV locus within a codon (PolyPhen2)	11
SeqCons	sequence conservation	12

### Extracting sequence-based features

We tested five sequence-based features that are specific to the prior loci-based information about the SNV that are not dependent on the difference between wild-type and altered nucleotide. The list of features is summarized in the second half of Table 2 and the details of the sequence-based feature extraction process are provided in the S2 Appendix.

### Predicting effects of SNV via Weka

After generation of the features, we trained and tested the features using Weka [29, 30], a data-mining software that contains multiple machine learning methods. Seven machine learning algorithms in Weka were used: naive Bayes, support vector machine (SVM), logistic regression, multi-layer perceptron (MLP), k-nearest neighbors (KNN), decision table, and random forest. For each of the algorithms and hyperparameter combinations, we feed Weka features and labels of 80% of HumVD-train. The hyperparameters of each machine learning algorithms were selected by validating on 20% of HumVD-Train (validation set). More specifically, we selected the hyperparameters that resulted in highest weighted F-scores over the validation set. Also, to accommodate for missing values of the test set, we used Weka’s unsupervised attribute filter to replace missing values.

### Performance comparison measures

We used weighted F-score to validate the performance of feature sets. The precision-recall based scores are known to be a measurement of choice when there is a class imbalance as in the case of our test sets. The weighted F-score is a weighted average of the precision and recalls that account for both the correctly predicted positives as well as incorrectly predicted positives and negatives. The value reaches its best value at 1 and worst at 0. Weighted F-score was also used to compare our methods with existing SNV prediction methods.

### Parameters used in comparative study

We compared the weighted F-scores of five features sets to nine SNV prediction methods as shown in the Table 3. Most of the methods are web-server based and take mutation location as input and output either numerical or categorical predictions. In the case of CADD, precomputed scores are provided. We tested on two threshold values 15 and 20, as suggested by the authors [31], to label SNV as Benign or Pathogenic.

**Table 3:** SNV prediction methods used in the comparative study. ‘Type’ represents whether the features are generated on a web-server or pre-computed. ‘Benign labels’ and ‘Pathogenic labels’ represent how classification labels of each algorithm was interpreted to match to Benign and Pathogenic labels.

Algorithms	Type	Benign labels	Pathogenic labels
PolyPhen2 [8]	web-server	benign	probably damaging, possibly damaging
SIFT [32]	code, precomput.	tolerated, t. low confidence	deleterious, d. low confidence
CADD [31]	precomput.	*X* < = threshold	*X* > threshold
FATHMM [33]	web-server	tolerated	damaging
LRT [34]	web-server, precomput.	neutral	damaging
M-CAP [35]	web-server	neutral	damaging
MutationAssessor [36]	web-server	low, neutral	high, medium
MutationTaster [37]	web-server	polymorphism, p. automatic	disease causing, d.c. automatic
PROVEAN [38]	web-server	neutral	damaging

## Results

### Classification performance between machine learning methods

We first provide the classification results utilizing seven machine learning method listed in the Method section 1.

#### Small difference in performance between learning algorithms

Classification accuracies of seven machine learning algorithms were measured to validate the effectiveness of the proposed loci-based features. The goal of the learning task was to classify an SNV site as pathogenic or benign based on the features defined at the SNV locus.

Table 4 shows the weighted F scores for each of the machine learning algorithms on the validation set (20% HumVD-train). The weighted F-scores on the validation set are used to selected hyperparameters for each machine learning algorithms. We observed that performance of algorithms was dependent more on the features and less on the algorithm used. That is, the difference of F-scores for each feature sets were small among learning algorithms. Although KNN had the highest F value of 0.91, the value of k varied for each feature sets. Thus we chose Random Forest that performed well across all feature sets and that depended less on the hyperparameters.

**Table 4:** Validation results for five features sets. Weighted F scores are reported on the validation set (20% HumVD-train) for all algorithms. ‘Sequence’ refer to sequence-based features (PSIC, Nobs, MinDJxn, CodPos and SeqCons); ‘Structure’ refers to structure-based features (KDmean, RSAmax, nRSA, nNum, Bstddev, nB, nKD, nSC, nBinding, Binding, Mapreg); ‘Str no Neigh’ refers to structure-based features without neighbour information (KDmean, RSAmax, Bstddev, Binding, Mapreg); ‘Seq + Str’ refers to all sixteen sequence and structure features listed above; ‘All + mutation’ refers to all prior features listed above and features based on difference between wild-type listed in S1 Table. The boldface numbers highlights top two weighed F scores for each feature sets.

Algorithm	Feature sets				
	Sequence	Structure	Str. noNeigh	Seq + Str	All + mutation
Naive Bayes	**0.84**	0.71	0.69	0.82	0.89
SVM	0.81	0.67	0.64	0.82	**0.90**
Logistic Regression	0.81	0.68	0.65	0.82	**0.90**
KNN	**0.84**	**0.73**	**0.73**	0.82	**0.91**
MLP	0.82	0.69	0.68	**0.83**	0.89
Decision Table	**0.83**	0.71	**0.72**	**0.83**	0.89
Random Forest	**0.83**	**0.74**	0.71	**0.85**	**0.90**

#### Structure-based features improve performance

**Fig 3:**
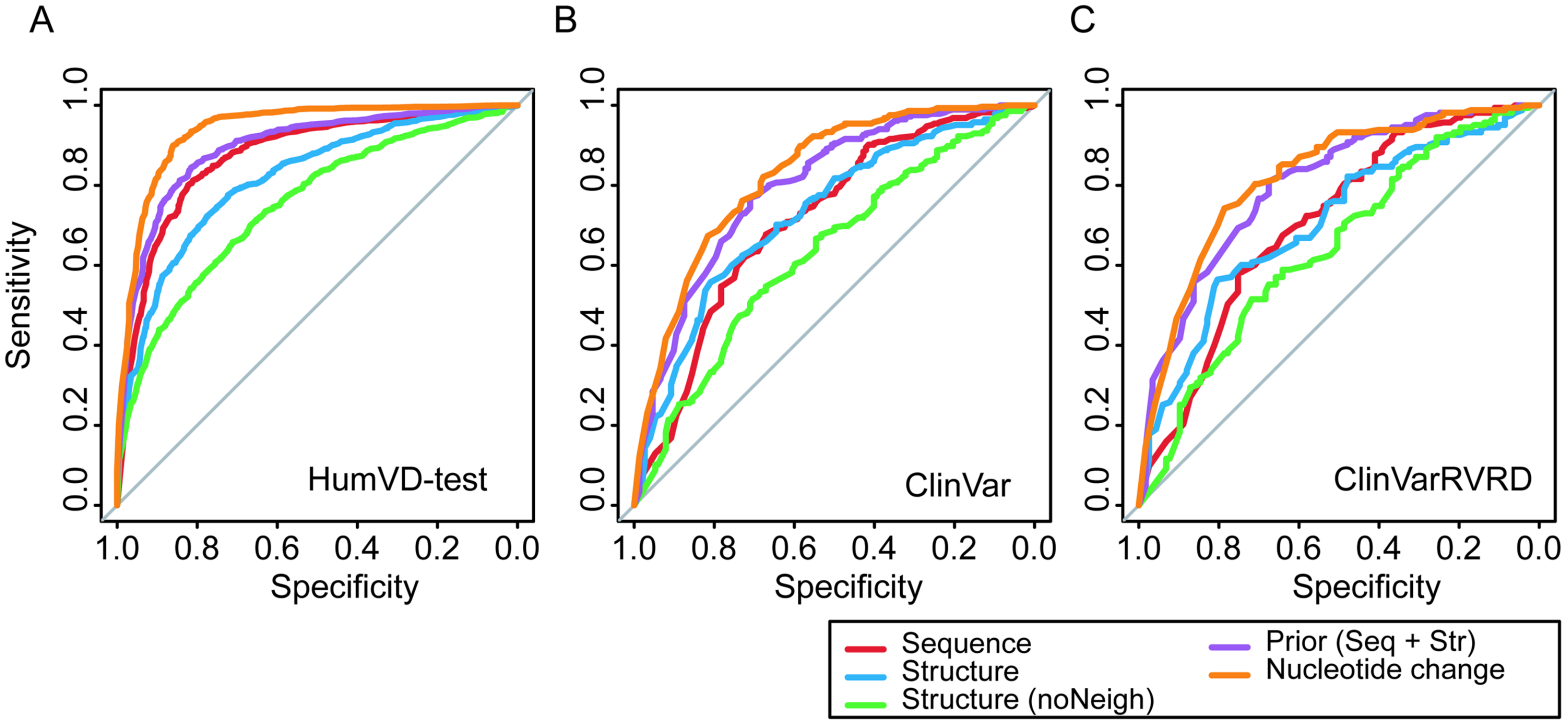
ROC curves for three datasets. **A**. HumVD, **B**. ClinVar, and **C**. ClinVarRVRD.

After selection of learning algorithm, Random Forest, we retrained the model using HumVD-train and tested the feature sets on three test datasets. Fig 3 shows the ROC curves for all test set and the performance comparison between features follows the same order. Also, the best-weighted F scores obtained by varying threshold in ROC for different feature types are shown in Table 5.

**Table 5:** Test results for different features. Summary of results for multiple feature types on optimal weighted F scores. (FNR = False Negative Rate, TPR = True Positive Rate, FPR = False Positive Rate, TNR = True Negative Rate).

Dataset	Feature set	FNR	TPR	FPR	TNR	Weighted F
HumVD test	Sequence	0.12	0.88	0.29	0.71	0.83
	Structure	0.21	0.79	0.29	0.71	0.76
	Structure (noNeigh)	0.17	0.83	0.50	0.50	0.72
	Prior (Seq + Str)	0.13	0.87	**0.22**	0.78	0.84
	All + mutation	**0.04**	**0.96**	**0.22**	**0.78**	**0.90**
ClinVar	Sequence	0.10	0.90	0.59	0.41	0.71
	Structure	0.18	0.82	0.50	0.50	0.70
	Structure (noNeigh)	0.21	0.79	0.62	0.38	0.64
	Prior (Seq + Str)	0.20	0.80	**0.34**	**0.66**	0.75
	All + mutation	**0.08**	**0.92**	0.45	0.55	**0.78**
ClinVarRVRD	Sequence	0.28	0.72	0.42	0.58	0.66
	Structure	**0.18**	**0.82**	0.52	0.48	0.67
	Structure (noNeigh)	0.41	0.59	0.36	0.64	0.61
	Prior (Seq + Str)	0.19	0.81	0.32	0.68	0.75
	All + mutation	0.20	0.80	**0.29**	**0.71**	**0.76**

On HumVD-test dataset, sequence features performed better than structure features. Interestingly the difference was minor for the ClinVar dataset with weighted F scores of 0.71 and 0.70 for sequence and structure, respectively. ClinVarRVRD’s structure features were slightly better than sequence features with F scores 0.67 and 0.66, respectively. However, prior sequence- and structure-based features performed better for all test datasets. Regarding individual features, six out top 50% ranked features are structure-based features as shown in the third column of Table 2. The result shows that, although the performance of structure-based features is similar or even lower, they are contributing regarding predicting the importance of loci in disease.

#### Neighborhood features improve predictions

We next tested the contribution of the neighborhood features in improving the classification performance. The performance of structure-based features with and without the neighborhood information was compared using ROC and weighted F scores. Neighborhood information improved the weighted F scores of learning algorithms for all test datasets (Table 5) and for all algorithms (Table 4). Regarding individual features, three out top 50% ranked features are structure-based features as shown in the third column of Table 2.

#### Structure-based features generalizes better

Compared to HumVD-test, weighted F scores decreased overall for ClinVar and ClinVarRVRD with the highest value being 0.78 compared to 0.90 for HumVD-test. The decrease was expected since ClinVar labeling and HumVD labeling criterion and the selection of benign cases are slightly different. With the difference in the distribution of training set and testing set in mind, we focused on the stability or generalization of the features across the datasets.

We observed that, although the model was trained on HumVD-train dataset, the differences in weighted F-scores of structure-based features were smaller within a difference of 0.09 (ranging 0.67 to 0.76) while differences in weighted F-scores of the sequence-based features were larger (ranging 0.66 to 0.83) when tested on new datasets, ClinVar and ClinVarRVRD.

### Performance comparison with existing methods

To compare the performance of our method with other algorithms, we performed a test on ClinVar and ClinVarRVRD dataset using weighted F-scores. Many of the existing methods listed in Table 6 includes the HumDiv and HumVar in their training. We did not test on HumDV-test, a subset of HumDiv and HumVar, since the methods will output biased results. Furthermore, since the existing algorithms utilized the difference between wild-type and variant in addition to the loci information, we compared our proposed features with or without selected features that utilize the difference between wild-type information. The difference between wild-type information used is listed in S1 Table.

**Table 6:** Performance comparison with existing methods.

Algorithm	Weighted F scores	
	ClinVar	ClinVarRVRD
PolyPhen2 [8]	0.73	0.69
SIFT [32]	**0.77**	0.70
CADD [31] (thresh = 15)	0.70	0.65
CADD [31] (thresh = 20)	0.75	0.70
FATHMM [33]	0.69	0.66
LRT [34]	0.73	0.71
M-CAP [35]	0.69	0.58
MutationAssessor [36]	0.68	0.64
MutationTaster [37]	0.55	0.44
PROVEAN [38]	0.71	0.69
Prior (Seq + Str)	0.75	**0.75**
All + mutation	**0.78**	**0.76**

Our comparisons found that existing algorithm’s performance varies with test data. They were able to perform well on ClinVar tests and our mutant features were marginally better. However, for Rare Variants (ClinVarRVRD), our prior features and mutant features performed better than existing algorithms.

### Structural examination of rare variants in rare disease cases

To understand the contribution of structure-based features, we took a detailed look at cases in the ClinVarRVRD dataset where structure-based features correctly determined the benign and pathogenic effects of SNV while sequence features failed. Examining cases that structure features correctly predicted as pathogenic while sequence features did not, we found many SNVs located at the binding regions or part of structurally stabilizing regions which are essential for maintaining overall structure of the protein. For the cases that structure-based features correctly predicted as benign while sequence features did not, we observed that SNV were located at regions that are relatively unstable or solvent accessible.

**Fig 4:**
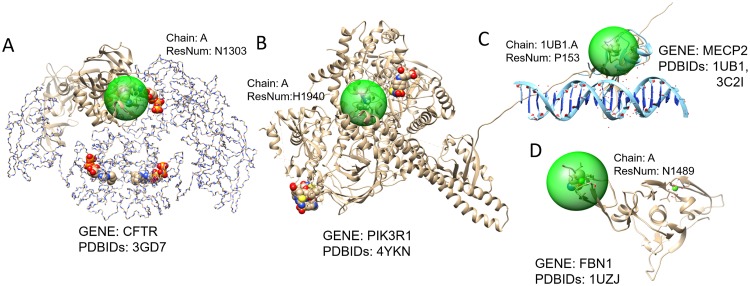
Pathogenic rare variants in binding sites. **A**. SNV at chr7:117590378 (Residue N1303) associated with a rare disease, Cystic fibrosis. The SNV locus is near the binding site of N6-Phenylethyl-ATP. **B**. SNV at chr5:68296301 (Residue H1940) associated with the SHORT syndrome. The SNV locus is a binding site compound 5e **C**. SNV at chrX:154031373 (Residue P153) associated with Rett syndrome. The SNV locus is near DNA binding site. **D**. SNV at chr15:48468527 (Residue N1489) associated with the Marfan syndrome. The SNV locus is at the calcium bind site.

We found in many cases if the SNV is at a binding site, the effect of the variant is pathogenic. Fig 4 shows four representative pathogenic SNVs at or near a binding site. SNV locus in Fig 4A is both an ATP binding site and a protein-protein binding site that is known to be associated with Cystic fibrosis. SNV locus in Fig 4B is near a lead (compound 5e) binding site that is known to be associated with the SHORT syndrome. SNV locus in Fig 4C is near DNA binding site that is known to be associated with Rett Syndrome. SNV locus in Fig 4D is directly at a calcium binding site.

**Fig 5:**
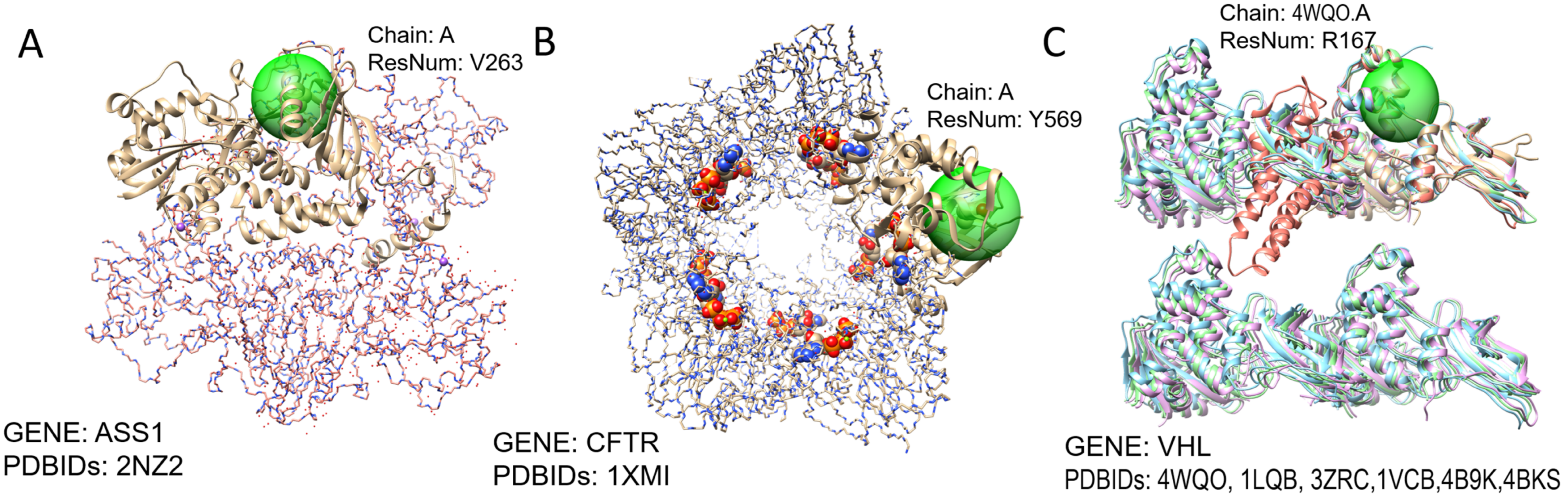
Pathogenic rare variants in structurally stable sites. **A**. SNV at chr9:130480398 (Residue V263) associated with a rare disease, Citrullinemia type I. The SNV locus is on a stable *α*-helix structure near domain binding site. **B**.SNV at chr7:117590378 (Residue Y569) associated with a rare disease, Cystic fibrosis. The SNV locus is in the middle of a stable triple-stranded parallel *β*-sheet. **C**.SNV at chr3:10149822 (Residue R167) associated with a rare disease, Pheochromocytoma. The SNV locus is part of a stable *α*-helix structure.

Also, many SNVs in structurally stable regions were pathogenic. Fig 5 shows three such cases. SNV locus in Fig 5A and 5C are parts of a stable *α*-helix structure. Where Fig 5A is also a domain binding region that forms a homo tetramer. Also, SNV locus in Fig 5B at a is in the middle of a stable triple-stranded parallel *β*-sheet of a homo 10-mer transmembrane structure near membrane binding area.

**Fig 6:**
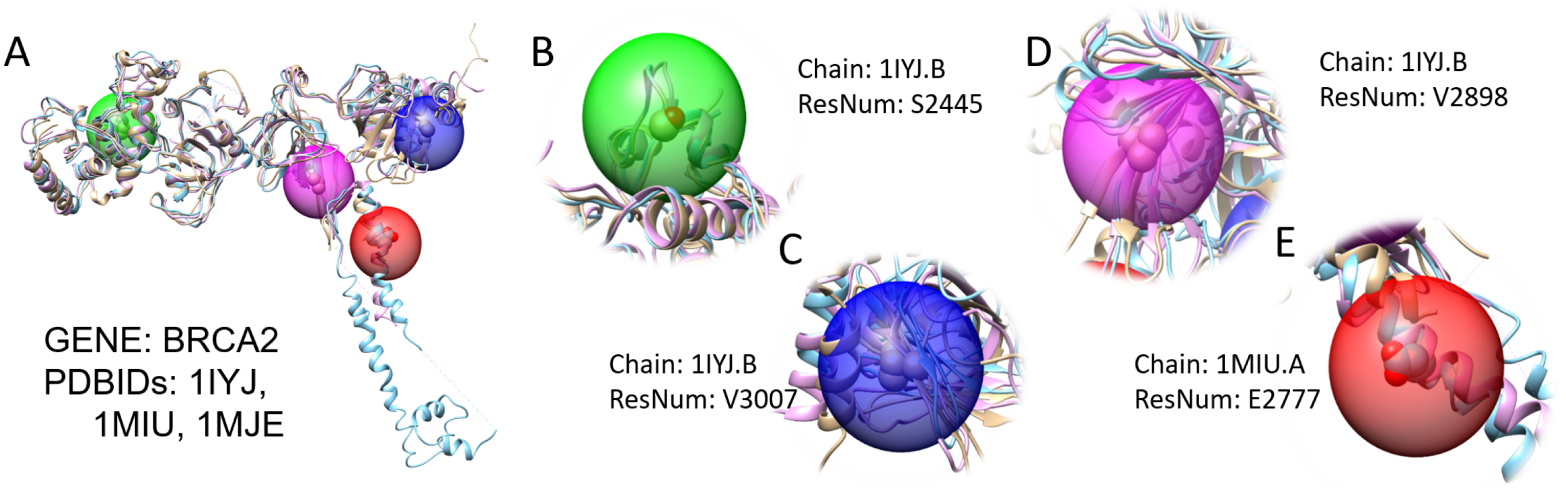
Four benign SNV on BRCA2 gene. **A**. Multiple alignment of PDBIDs:1IYJ,1MIU, and 1MJE marked with four benign SNV loci. **B**. SNV at chr13:32356536 (Residue S2445) tip of a short *α*-helix. **C**. SNV at chr13:32380124 (Residue V3007) tip of unstable *β*-sheet. **D**. SNV at chr13:32379467 (Residue V2898) middle of unstable *β*-sheet. **E**. SNV at chr13:32371035 (Residue E2777) middle of unstable *α*-helix where one of the structure, PDBID:1IYJ, terminates.

For benign cases, we looked at four benign SNVs on BRCA2 gene as shown in Fig 6. All of the four loci are predicted to be pathogenic by sequence-based features alone. However, structure-based features predicted them as benign, following the ClinVar annotation. We observed that these locations were mostly solvent accessible and inconsistent secondary structure assignments were observed in the positions among homologous structures.

## Discussions and conclusions

Considering that the number of rare variants in rare diseases cases is inherently limited, it is important to be able to extract information that is less dependent on the number of known cases. Protein structure provides direct information about the functional importance of loci and does not rely heavily on statistical means for prioritizing the importance of mutation loci. These structure-based features need to be extensively tested, especially when several lines of empirical evidence demonstrate the presence of causal mutations at critical structural positions.

Loci based prior features performed comparably with features based on the difference between wild-type and variant, which suggests the importance of SNV loci. Amongst the prior features, our results show that sequence-based features perform slightly better than structure-based features in classifying pathogenic SNVs. However, structure-based features, when combined with sequence-based features, improved the weighted F scores in all three test datasets. Regarding structure-based features, we made two novel contributions that have been shown to improve the prediction performance: use of multiple homologous structures and use of features from the structural neighborhood. The structural neighborhood improved the overall prediction in all test datasets. Also, empirical structure-based features of a rare variant in rare disease cases showed that structure-based features play an important role in correctly classifying benign versus pathogenic cases by identifying binding site information and structurally stable information. We also compared our proposed features with numerous existing methods and showed increased performance. All of the methods, PolyPhen2 [8], SIFT [32], CADD [31], FATHMM [33], LRT [34], M-CAP [35], MutationAssessor [36], MutationTaster [37], and PROVEAN [38], although most use structure-based features, do not focus on the careful generation of structural features. We believed that generation of better structure-based features will be able to improve theses methods as well.

We do recognize the limitation of structure-based features, such as limited coverage. However, we believe that the limitations can be addressed via various computational prediction methods, such as structure prediction in the presence of mutation [39], reconstruction of protein-protein interactions [40], ligand binding site predictions [41], and stability predictions [42]. In the future, we would like to include more structure-based features utilizing computational methods such as binding site predictions, and stability predictions and also increase coverage by including structure prediction methods.

## Supporting information

S1 Appendix Constructing structure-based features. (PDF)

S2 Appendix Constructing sequence-based features. (PDF)

S1 Table Difference between wild-type based features. Each of features are obtained from PolyPhen2 server (URL: http://genetics.bwh.harvard.edu/pph2/dokuwiki/appendix_a).
(PDF)

S1 File HumVar-HumDiv training set. List of HumVar-HumDiv training set features and meta data.
(CSV)

S2 File HumVar-HumDiv test set. List of HumVar-HumDiv test set features and meta data.
(CSV)

S3 File ClinVar data set. List of ClinVar test set features and meta data.
(CSV)

S4 File ClinVarRVRD data set. List of ClinVarRVRD test set features and metadata.
(CSV)
