# Supplementary material for: Impact of structural prior knowledge in SNV prediction: Towards causal variant finding in rare disease
Source: PLoS One. 2018 Sep 28;13(9):e0204101. doi: 10.1371/journal.pone.0204101 (PMC6161878; doi:10.1371/journal.pone.0204101)
Supplement: S1 Appendix — (PDF) [file pone.0204101.s001.pdf]

S1 Appendix. Constructing Structure-Based Features Another novelty of our structure-based feature is on the extraction of information from multiple homologous structures. The reason why we have decided to take information from multiple structures is that a PDB structure is only a static snapshot of the true protein and the functional information such as binding event may exist in one structure while not on another depending on the experimental settings, thus examining all homologous structures is important. Again, the eleven structure-based features depend on the location of the SNV and not on the actual difference between wild-type and are considered prior knowledge.

The first structure-based feature is the binding site information (*Binding*). The binding feature was generated by finding ligands, ions, and other biomolecules that were within a threshold distance to the protein structure. For small molecules, i.e., ion binding site and protein-ligand binding site, we used a threshold of 4.5Å. For larger binding targets, i.e., DNA, RNA, and protein-protein binding sites, we used a threshold of 8Å. The thresholds were chosen to represent the approximate distance between the bound structures found in PDB database (http://www.rcsb.org/). If a target molecule was observed within the threshold, then the corresponding binding site described as a tuple of size five (ion, ligand, DNA, RNA, and protein) was set to 1. Binding information from multiple structures was integrated using binary *OR* operation to represent all cases of possible binding sites at the query site among multiple structures.

Based on the five binding target information, i.e., ion, ligand, DNA, RNA, and other protein, extracted from the structure, we summed the values such that the resultant feature represents the number of different binding types found at the query site. Binding information for the neighborhood (*nBinding*) was calculated as the number of different binding types found in the structural neighbors.

The second type of structure-based feature is hydrophobicity (*KDmean*). We computed *KDmean* as the mean value of Kyle Doolittle (KD) hydrophobicity [1] values of amino acids observed at the query location from multiple aligned structures. The mean KD hydrophobicity value of the structural neighborhood was also used (*nKD*).

The third type of feature is the solvent accessibility. Solvent accessibility is the surface area which is reachable by a solvent in squared Angstroms. DSSP [2,3] was used to calculate the initial solvent accessibility. After which the accessibility value was divided by the maximum exposure value possible for the amino acid triplets centered around the mutation point to obtain the Residual Solvent Accessibility (RSA) value. The maximum RSA value (*RSAmax*) was chosen as a feature to represent information from multiple structures. Mean of the maximum RSA values of amino acids in the neighborhood (*nRSA*) was also used.

Fourth feature type is B factor. B factor is a temperature dependent parameter which describes the displacement of atomic positions from their mean position that indirectly accounts for the flexibility of the locus. This feature for a given amino acid was obtained directly from the PDB files. We use the standard deviation of B-factor (*Bstddev*) values to represent the variability of this parameter in multiple structures as a feature and the mean B-factor value of amino acids in the neighborhood (*nB*) as the neighborhood feature.

Fifth feature type was calculated based on neighborhood sequence conservation. The sequence conservation of the locus was considered as a sequence-based property, and the method of calculating this value is described in the next section. However, since the neighborhood is a structurally defined concept, and mean of sequence conservation of amino acids in the neighborhood (*nSC*) was included as a structure-based feature.

The last structure-based feature type comes from the Ramachandran plot. The Ramachandran plot depicts the phi-psi angles of amino acids in a given protein structure. A Ramachandran plot was partitioned into regions where a secondary

structure was more likely to be found. To generate related feature, we used PolyPhen2 web server to get MapReg feature for a locus. Based on the MapReg feature generated, we represented our feature as a binary value (*Mapreg*) describing whether the mutation site belongs to core regions (represented by symbols A, L, B, P), or not.

References

1. Kyte J, Doolittle RF. A simple method for displaying the hydropathic character of a protein. Journal of molecular biology. 1982;157(1):105–132.

2. Kabsch W, Sander C. Dictionary of protein secondary structure: pattern recognition of hydrogen-bonded and geometrical features. Biopolymers. 1983;22(12):2577–2637.

3. Touw WG, Baakman C, Black J, te Beek TA, Krieger E, Joosten RP, et al. A series of PDB-related databanks for everyday needs. Nucleic acids research. 2015;43(D1):D364–D368.
