# Supplementary material for: Impact of structural prior knowledge in SNV prediction: Towards causal variant finding in rare disease
Source: PLoS One. 2018 Sep 28;13(9):e0204101. doi: 10.1371/journal.pone.0204101 (PMC6161878; doi:10.1371/journal.pone.0204101)
Supplement: S2 Appendix — (PDF) [file pone.0204101.s002.pdf]

S2 Appendix. Constructing Sequence-Based Features We tested five sequence-based features that are specific to the prior loci-based information about the SNV and are not dependent on the difference between wild-type. The first feature is the sequence conservation (*SeqCons*). The sequence conservation implies conservedness of the locus throughout the evolution process. The assumption being that the locus that is unchanged across species must be functionally important. We computed the sequence conservation by calculating the variation of amino acids in loci. That is, after multiple sequences aligning via ClustalW [1, 2] the query sequence with the homologous sequence extracted from MetaPhOrs API [3], a variation of an amino acid at a mutation point was calculated and conservation score was computed using the Jensen-Shannon divergence. Four additional features were adapted from sequence-based features of PolyPhen2 from DNA and protein levels. At the DNA level, the position SNV locus within the codon (*CodPos*) and the distance of the SNV locus from the closest intron/exon junction (*MinDJxn*) were identified and included as a sequence-based feature. At the protein level, Position-Specific Independent Counts (*PSIC*) score [4] of the wild-type amino acid residue and the number of amino acids observed at SNV locus (*Nobs*) during multiple sequence alignment of homologous sequences were used.

References

1. Larkin MA, Blackshields G, Brown N, Chenna R, McGettigan PA, McWilliam H, et al. Clustal W and Clustal X version 2.0. bioinformatics. 2007;23(21):2947–2948.

2. Goujon M, McWilliam H, Li W, Valentin F, Squizzato S, Paern J, et al. A new bioinformatics analysis tools framework at EMBL–EBI. Nucleic acids research. 2010;38(suppl 2):W695–W699.

3. Pryszcz LP, Huerta-Cepas J, Gabaldón T. MetaPhOrs: orthology and paralogy predictions from multiple phylogenetic evidence using a consistency-based confidence score. Nucleic acids research. 2010; p. gkq953.

4. Sunyaev SR, Eisenhaber F, Rodchenkov IV, Eisenhaber B, Tumanyan VG, Kuznetsov EN. PSIC: profile extraction from sequence alignments with position-specific counts of independent observations. Protein engineering. 1999;12(5):387–394.
