# Supplementary material for: Impact of structural prior knowledge in SNV prediction: Towards causal variant finding in rare disease
Source: PLoS One. 2018 Sep 28;13(9):e0204101. doi: 10.1371/journal.pone.0204101 (PMC6161878; doi:10.1371/journal.pone.0204101)
Supplement: S1 Table — Each of features are obtained from PolyPhen2 server (URL: http://genetics.bwh.harvard.edu/pph2/dokuwiki/appendix_a). (PDF) [file pone.0204101.s003.pdf]

S1 Table. Difference between wild-type based features. Each of features are obtained from PolyPhen2 server (URL: http://genetics.bwh.harvard.edu/pph2/dokuwiki/appendix_a).

Feature	Description
Score2	the PSIC score for mutant amino acid
dScore	represents the difference *PSIC* and *Score2*
Transv	represents if the substitution is a transversion
CpG	values represent change in CpG content due to substitution
IdPmax	represents mutant amino acid's maximum congruency in multiple sequence alignment
IdQmin	represents the sequence identity to closest homologue deviating from wild type amino acid
